# The splicing genes *SmEa *and *SmEb* regulate plant development during vegetative growth in poplar

**DOI:** 10.1186/s12870-025-07676-3

**Published:** 2025-12-23

**Authors:** Daniela Goretti, Silvio Collani, Alice Marcon, Ove Nilsson, Markus Schmid

**Affiliations:** 1 Department of Plant Physiology, Umeå Plant Science Centre, Umeå University, Umeå, SE-90187 Sweden; 2 Department of Forest Genetics and Plant Physiology, Umeå Plant Science Centre, Swedish University of Agricultural Sciences, Umeå, 901 83 Sweden; 3 Department of Plant Biology, Linnean Center for Plant Biology, Swedish University of Agricultural Sciences, Uppsala, S-75007 Sweden; 4 DISSTE, University of Eastern Piedmont, Vercelli, 13100 Italy; 5 Department of Plant Developmental Biology, Max Planck Institute for Plant Breeding Research, Cologne, 50829 Germany

**Keywords:** Splicing, Poplar, Development, Sm, CRISPR/Cas9

## Abstract

**Background:**

Spliceosomes are large evolutionary conserved ribonucleoprotein complexes containing at their core heptameric rings of Sm (or LSm) proteins and U-rich snRNAs. The role of Sm proteins in animal development is well established, and recent research has begun to link mutations in these genes to growth defects in plants. One of the most studied *Sm* genes is *SmE1/PCP*, mutants of which display a temperature-dependent phenotype in *Arabidopsis* thaliana.

**Results:**

This study provides a first glimpse into the function of a core splicing protein in the regulation of growth in a perennial species. Phylogenetic analysis identified two paralogous *SmE* genes in poplar, named *SmEa* and *SmEb*, that encode identical proteins and are orthologs of *SmEs* from *Arabidopsis*, as suggested by Y2H and in vivo experiments. CRISPR/Cas9 mutagenesis in hybrid aspen identified a role for *SmEs* in development in plants grown in an environment simulating seasonal photoperiod and temperature changes. Unlike in Arabidopsis, low temperatures had no or only a very minor effect on the development of *sme* mutants in aspen.

**Conclusions:**

We identified specific aspects of *SmE* in poplar, highlighting the importance of examining the physiological and evolutionary differences that define this gene family in woody compared to herbaceous plants.

**Supplementary Information:**

The online version contains supplementary material available at 10.1186/s12870-025-07676-3.

## Introduction

Spliceosomes are evolutionarily conserved ribonucleoprotein complexes common to the eukaryotic kingdom and are composed of U-rich small nuclear RNAs (snRNAs) and associated Sm or LSm protein heptameric rings. This Sm/LSm ring provides a structural framework, and is essential for the biogenesis, stability, specificity, and function of snRNPs in pre-mRNA splicing [1]. While the role of Sm proteins is well characterized in animal systems, where they are indispensable in regulating processes from early embryogenesis to tissue differentiation, our understanding of how they influence development in plants starts to emerge.

In the model plant *Arabidopsis thaliana*, one of the most investigated *Sm* genes is *SmE1*, also known as *PORCUPINE* (*PCP*). Mutations in this gene lead to a striking conditional phenotype: when grown at lower ambient temperatures, mutants exhibit stunted shoot growth coupled with aberrant leaf development and defects in root architecture [2–4]. This temperature-sensitive phenotype suggests that SmE1 plays a critical role in mediating splicing robustness under cooler conditions.

Gene expression analysis in the *sme1-1/pcp-1* mutant identified induction of stress-response pathways as a possible explanation for their stunted shoot phenotypes. Specifically, transcriptome analyses showed upregulation of genes associated with cold stress, osmotic stress, and general defence responses under cool but non-stressful ambient temperature (16 °C), but did not detect widespread misregulation of developmental genes that might account for the pleiotropic defects observed in the *sme1-1/pcp-1* mutant. These findings suggest that the loss of PCP triggers an exaggerated stress response under normally permissible temperatures which may underlie the observed temperature-specific growth impairment in the *sme1-1/pcp-1* mutant [5].

More recently, several lines of evidence have implicated disturbances in hormone homeostasis in the root phenotypes in *sme1-1/pcp-1* mutants. In particular, auxin, which is well known for its central role in governing root patterning, cell division, and elongation, has been implicated in the *sme1-1/pcp-1* root phenotype [6]. Aberrant localization or metabolism of auxin could disrupt gradient formation necessary for root meristem maintenance and directional growth, providing a plausible mechanistic link between splicing defects and developmental anomalies.

Despite these advances in *Arabidopsis*, the role of core splicing factors in shaping growth and form in perennial species remains largely unexplored. Understanding how *SmE* genes contribute to the development of long-lived woody plants such as *Populus* is not only of basic biological interest but also bears potential significance for forestry and climate resilience. As perennials often endure repeated cycles of environmental fluctuation throughout their lifespan, ensuring splicing fidelity may be especially critical in these systems to maintain developmental stability and adaptability.

Accordingly, in this study we characterize *SmE* in poplar, assessing the expression patterns, phylogenetic relationships, and functional impact, particularly under variable temperature regimes. By leveraging comparative genomics, transcriptional profiling, and reverse genetic approaches, we elucidate Sm function in poplar. Through this work, we seek to advance our understanding of how fundamental splicing machinery contributes to developmental robustness across plant lifespans and environmental contexts.

## Materials and methods

### Plant material

Hybrid aspen (*Populus tremula* x *Populus tremuloides*) clone T89 was used as wild-type control and as background for CRISPR/Cas9 mutagenesis [7]. Plants were cultivated on ½ strength MS medium until rooted. Once on soil, plants were grown in growth chambers in long day (LD; 16 h light, 21 °C/6 h dark, 21 °C) and with weekly fertilization (10 mL NPK-Rika S/plant). Illumination was from ‘Powerstar’ lamps (HQI-T 400 W/D BT E40, Osram, Germany) giving an R/FR ratio of 2.9 and a light intensity of 150–200 mmol m-2 s-1. To induce growth cessation, plants were moved to short day (SD; 10 h light, 21 °C/14 h dark, 21 °C) and fertilization was stopped. For dormancy release, plants were treated with cold (8 h light, 4 °C/16 h dark, 4 °C) for 7 weeks and then transferred back to LD for bud flush. In both SD and LD, previously published bud scores were used to assess the effects on bud development (set/flush) [8]. For year-around gene expression analysis, RNA from a previous study, which had sampled a ca. 40-year-old local (Umeå, Sweden) aspen tree (identified by Ove Nilsson) twice a month around midday, was used [9]. No permissions were required to collect these samples as the sampled tree was grown on university grounds, and no voucher specimen of this material has been deposited in any publicly accessible herbarium. *Arabidopsis thaliana*, accession Col-0 was used as a wildtype in this study. s*me1-1/pcp-1* is T-DNA insertion mutant originating from the Salk population [10] and has been previously described [2].

### Phylogenetic analysis

For the selection of *Sm* and *LSm* genes, we used genomic data from *P. tremula* available at PopGenIE. Coding regions were identified through BLAST searches against the mRNA data, using Sm/LSm CDS from *Arabidopsis* as queries. In cases where database annotations were inaccurate, manual curation was performed. Translated CDS from *P. tremula* and *Arabidopsis* genes were subsequently used in CLC Main Workbench (version 23.0.1) for phylogenetic analysis, employing the neighbor-joining (NJ) method with the Jukes–Cantor substitution model and 1000 bootstrap replicates.

### Generation of poplar *SmE* CRISPR/Cas9 lines 

Potential sgRNAs specifically targeting *PtSmEa* and *PtSmEb* were identified using E-CRISP (http://www.e-crisp.org/) and plasmids for plant transformation were generated as reported in André et al. (2022) [9]. Briefly, sgRNA sequences were introduced into entry vectors by PCR-based site-directed mutagenesis and the final vector (containing promoter, Cas9 CDS, terminator, two sgRNAs and resistance cassette) was assembled by GreenGate reaction (150 ng of each component, 1.5 µL FastDigest buffer, 1.5 µL of 10mM ATP, 1 µL 30U/mL T4 ligase, and 1 µL Eco31I in a 15 µL reaction) in 50 cycles of 5 min restriction/ligation at 37 °C and 16 °C, respectively, followed by 5 min 50 °C, and 5 min 80 °C [11]. *Escherichia coli* strain DH5a was used for amplification of all plasmids, which were then confirmed by sequencing (Eurofins). Vectors with different combinations of gRNAs (Table S18) were transformed into T89 using a standardized protocol [7]. At least 30 individual transgenic lines from each transformation were screened for target gene deletions (Fig. S2).

### Complementation of Arabidopsis *sme1-1/pcp-1* with *PtSmEb*

The *SmEb* coding sequence from *P. tremula* was amplified by PCR from cDNA and cloned using the GreenGate system [11]. The final construct was transformed into the *Agrobacterium tumefaciens* strain GV3101 containing the pMP90 and pSoup helper plasmids by electroporation (Gene Pulser Xcell system). Arabidopsis *sme1-1/pcp-1* mutant plants were transformed by the floral dip method. BASTA selection (0.1%, v/v) was used for screening the transgenic lines on soil. Lists of the PCR primers used for cloning can be found in Table S18.

### Construct generation and yeast-two-hybrid (Y2H) assay

For testing protein-protein interactions in a Y2H assay, the coding sequences of *SmE*, *SmF*, and *SmG* from *P. tremula* were cloned into the yeast vectors pGADT7 or pGBKT7 modified for the GreenGate cloning system [12]. Oligos used in the cloning are listed in Table S18. Pairs of vectors, including negative controls, were used to co-transform yeast strain AH109 and colonies carrying both vectors were selected on SD medium without tryptophan (-W) and leucine (-L) (TaKaRa 630317) at 28 °C. After 6 days, protein-protein interactions were tested by growing serial dilutions on SD drop-out medium lacking tryptophan (-W), leucine (-L), and histidine (-H) (TaKaRa 630319).

### Reverse transcription quantitative PCR (RT-qPCR)

Total RNA was extracted from leaves using the Qiagen Plant RNeasy kit and treated with RNase-free DNaseI (Thermo Scientific) to remove DNA contamination. cDNA was synthesized using the RevertAid First Strand cDNA Synthesis kit (Thermo Fisher) in accordance with the manufacturer’s instructions. RT-PCR was carried out using a CFX96 Real-time System (Biorad) and SYBR Green Master Mix (Bioline). The relative expressions were calculated using the 2^(−ΔΔCT)^ method. For each sample, three biological and three technical replicates were used. Primers used in RT-qPCR are listed in Table S18 and were designed to be able to amplify both genomes (*P. tremula* and *P. tremuloides*) in T89.

### RNA-Seq and data preprocessing

To analyse genes expression the first fully expanded leaf was sampled from T89 (control), *smea_26* and *smeb_3* at the end of the LD growth period, before the shift to SD conditions. One leaf per plant was sampled and a total of 4 bioreps were used (4 plants per line). Frozen samples were ground to a fine powder, and total RNA was extracted with Qiagen Plant RNeasy kit according to the manufacturer’s instructions and treated with DNAseI (Thermo Scientific). Strand-specific mRNA-Seq was conducted by Novogene using NEB Next^®^ Ultra RNA Library Prep Kit for Illumina and libraries sequenced on Illumina NovaSeq 6000 S4 flowcell with PE150.

Raw sequencing reads were filtered for residual ribosomal RNA (rRNA) contamination by using SortMeRNA (v4.3.4; [13] settings --log --paired_in --fastx–sam --num_alignments 1) and the rRNA sequences provided with SortMeRNA. Non-rRNA reads were then trimmed for sequencing adaptors and filtered for quality by using Trimmomatic (v0.39; [14] settings TruSeq3-PE-2.fa:2:30:10 SLIDINGWINDOW:5:20 MINLEN:50). Read quality was assessed before and after rRNA removal and quality filtering by using FastQC (http://www.bioinformatics.babraham.ac.uk/projects/fastqc/). Filtered reads were pseudo-aligned to *P. tremula* transcriptome (v2.2, obtained from PopGenIE; [15] using Salmon (v1.9.0; non default settings: --gcBias --seqBias) [16].

### Differential expression analysis, gene ontology enrichment and splicing analysis

Per-gene read counts from Salmon were imported in R (v4.3.1; R Core Team 2023) and normalized using a variance stabilizing transformation as implemented in DESeq2 (v1.42.1) [17]. Similarity within biological replicates was assessed by using custom R scripts, available from https://github.com/nicolasDelhomme/poplar-CRISPR-WGS. Differential expression was performed using DESeq2 with FDR-adjusted *p*-values threshold at 0.01, by comparing CRISPR-edited lines to the WT line. Gene ontology (GO) enrichment was performed using topGO with FDR-adjusted *p*-values threshold at 0.01. The applied fold-change threshold was set at 0.5. *P*-values were adjusted using the Benjamini-Hochberg procedure.

For splicing analysis, RNA-seq samples were aligned to the *Populus tremula* v2.2 genome reference using STAR v.2.7.9a. Differentially spliced transcripts between the mutants *smeb_3* and *smea_26* and wild-type T89 were found using the R-package ASpli. As default from the ASpli package, a bin FDR of 5%, a Junction FDR of 1% and a bin inclusion of 20% were applied. Local splicing events in the reference genome were annotated using SUPPA2 v. 2.3 (generate Events -e SE SS MX RI FL -f ioe). Only events annotated as Alt 3’, Alt 5’, IR, and ES were included as “Total Annotated”. In total, there were 19,297 local splicing events from these categories in the genome. There were in total 2,038 differential splicing events affected for *smeb_3* and 1,915 events for *smea_26* lines when compared to wild-type T89. Only looking at the event types Alt 3’, Alt 5’, IR, and ES (including events that were present in at least one isoform, represented by Aspli with *), there were 1,527 and 1,384 splicing events for mutants *smeb_3* and *smea_26* respectively.

Nucleotide sequences from the 5’ donor and 3’ acceptor splice sites of 467,737 introns in the *P. tremula* genome were extracted and used as input for creating pictogram logos with MEMESuite v. 5.5.2 (settings: -dna -nmotifs 1 -minw 5 -maxw 60 -mod anr). The same procedure was done for all differential IR events in the mutants (1,199 and 1,029 for *smeb_3* and *smea_26*, respectively). All scripts can be found in the public repository 10.5281/zenodo.14892044.

### WGS and off-targets analysis

The genomic DNA reads were aligned to Potra v2.2 and T89 v2.0 by using BWA-mem (0.7.17) and samtools (1.16). Duplicated reads were marked using Picard (2.27.1) and samtools (1.16). Variant (both SNPs and indels shorter than 50 bp) calling and filtering was first done using GATK (4.2.6.1). Subsequently, a second snp/indel call was made using bcftools to confirm the results, as the performance of GATK drops dramatically if the reference sequence consists of many short contigs, which is the case with the T89 genome. We used quite relaxed criteria for bcftools (--min-MQ 10 --min-BQ 20). The PAM sites were found using Cas-OFFinder (v2.4.1), where we tested allowing for 1, 3, and 5 mismatches between the PAM patterns (5’-NGG-3’/5’-NAG-3’) and the gRNA query sequences. The potential off-target sites reported by Cas-OFFinder were intersected with the SNPs/indels (which were expanded +/- 50 bp) using bedtools intersect (2.30.0) to find putative off-targets. Sequences for the Sm and LSm genes were retrieved from Potra v2.2 and blasted against the T89 v2.0 assembly using NCBI Blast+ (2.13.0). Only hits longer than 1kbp were kept. We investigated if the putative off-targets were close to or overlapping these genes. The transcript reads were aligned onto Potra v2.2 using STAR. The visualization was done using IGV.

The assumption made in this analysis is that a putative off-target is a site likely near a gene that closely matches a gRNA with a PAM immediately adjacent to it, where we observe an insertion or deletion. Decision tree for off-target classification: Indels/SNPs (Yes) ->Overlap gRNA with a PAM (Yes) ->Is it within/next to a gene (Yes) = > putative off-target.

## Results

### Identification of *SmE* genes in poplar

**Fig. 1:**
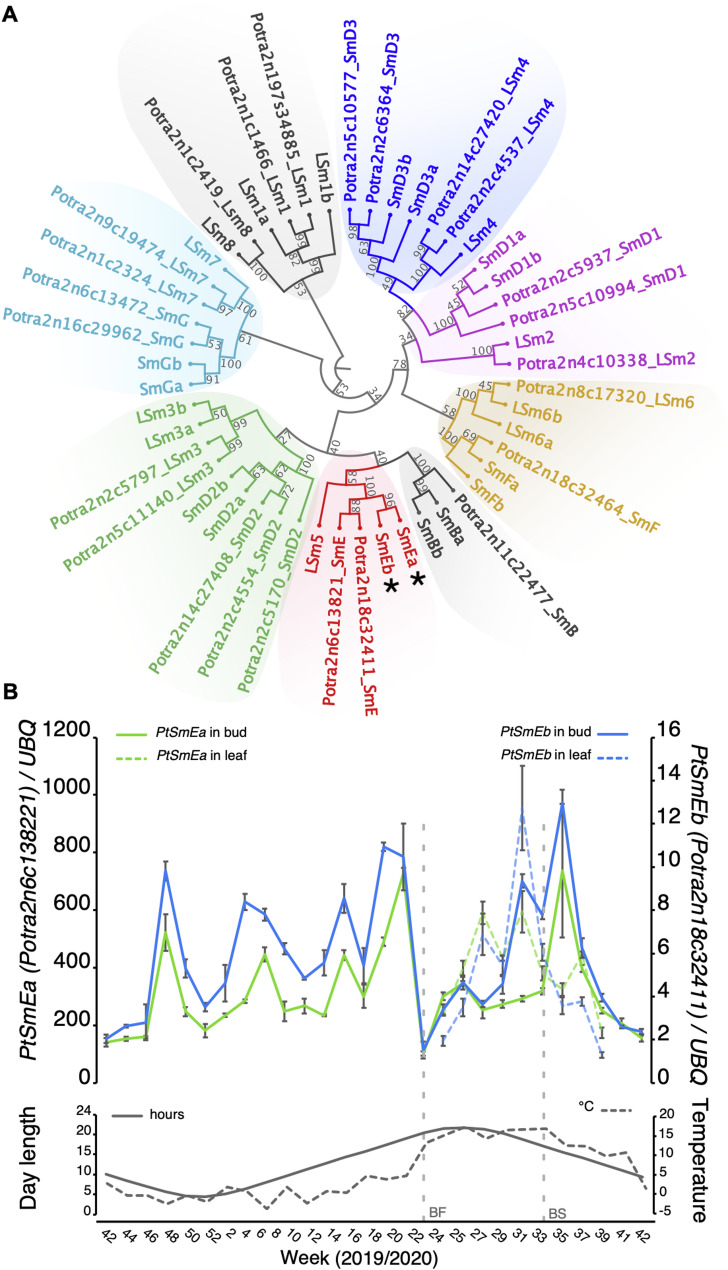
Identification of *PtSmEa* and *PtSmEb*. (**A**) Dendrogram depicting the phylogenetic relationship between Sm and LSm proteins in *Arabidopsis* (named Sm or LSm) and *Populus tremula* (named Potra_Sm or Potra_LSm). The tree was constructed using the Neighbor Joining method and the Jukes-Cantor model. Bootstrap analysis was performed with 1,000 replications, and support values are reported at each node. (**B**) Upper panel: Expression of *PtSmEa* and *PtSmEb* over one year (from October 2019 to October 2020) in tissues sampled at 2 pm from a field-grown *P. tremula* in Umeå (North Sweden). Error bars indicate s.d. of three biological replicates. Lower panel. Average day length and temperature per week. BF: bud flush; BS: bud set

As information about the role of core splicing genes in regulating development in perennial plants is essentially missing, we analysed *SmE* genes in aspen. Similar to *Arabidopsis*, most *Sm* and many of the *LSm* genes are duplicated in European aspen (Fig. 1A, Table S1). However, whereas the two *Arabidopsis* SmE protein paralogs SmE1/PCP and SmE2/PCP-Like are distinguished by two amino acid substitutions, the proteins encoded by the two *SmE* genes (*PtSmEa*, *Potra2n18c32411*; *PtSmEb*, *Potra 2n6c13821*) in *P. tremula* are identical (Fig. 1A, Fig. S1). *PtSmEa* and *PtSmEb* exhibit similar expression patterns in buds and leaves in field-grown mature *P. tremula* throughout the year, which is not surprising given that SmE is a core component of the spliceosome. The similarity of *PtSmEa* and *PtSmEb* expression patterns suggests the existence of a regulatory process controlling both genes throughout the year (Fig. 1B).

**Fig. 2:**
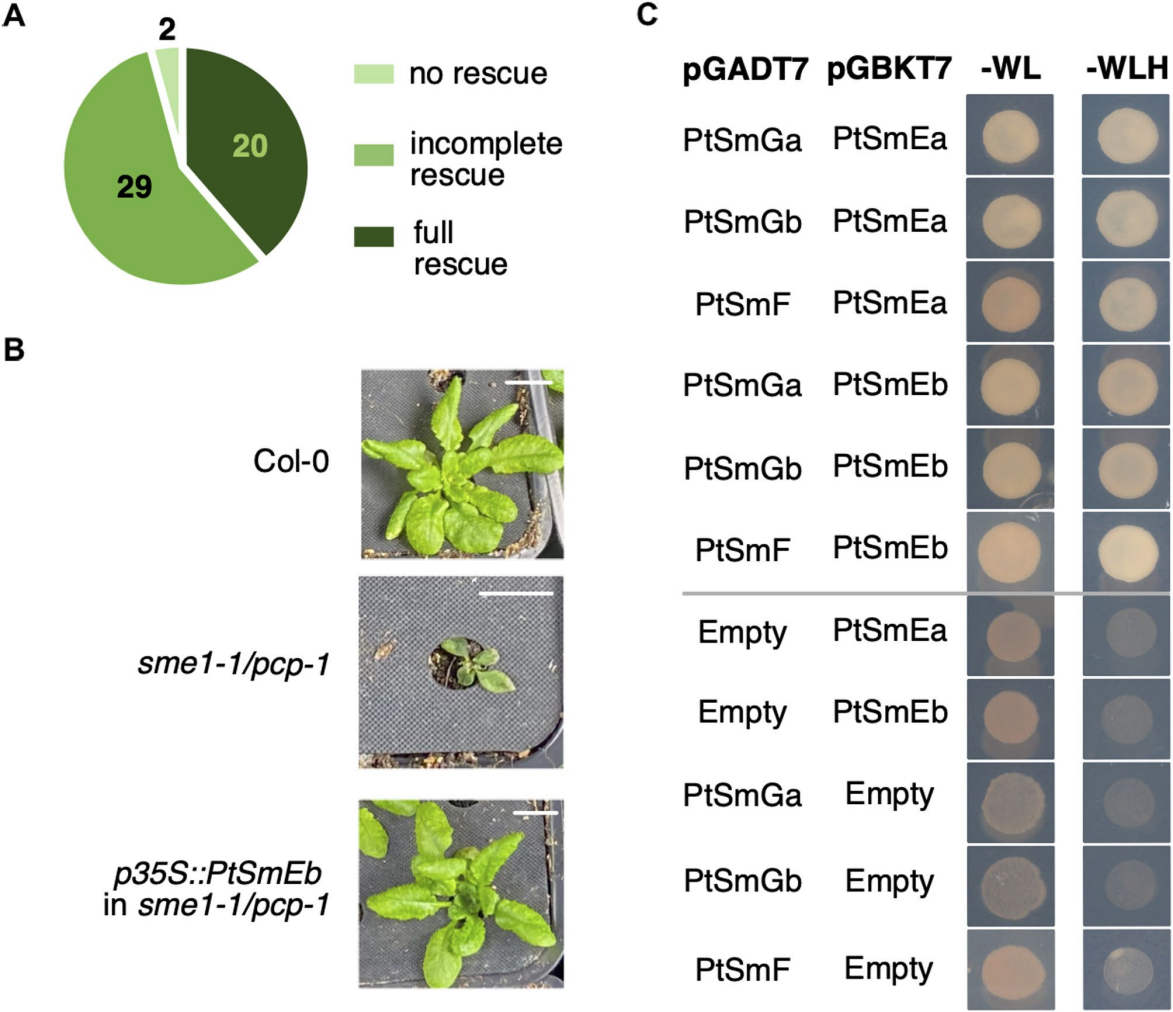
*PtSmEa* and *PtSmEb* are orthologues of *SmEs* from *Arabidopsis*. (**A**) Pie chart showing the number of T1 *sme1-1/pcp-1* plants expressing *p35S::PtSmEb* grown at 16 °C that display no, partial, or complete rescue. (**B**) Expression of *p35S::PtSmEb* rescues mutant phenotype of *sme1-1/pcp-1* when grown at 16 °C. Photos show a 42-day-old T1 plant expressing *p35S::PtSmEb* in *sme1-1/pcp-1* grown at 16 °C and control *sme1-1/pcp-1* and Col-0 plants of the same age. Size bar: 1 cm. (**C**) Interaction of *P. tremula* proteins was determined by yeast-2-hybrid assay. Sm proteins were cloned into the pGADT7 and pGBKT7 vectors, which provide in-frame fusions to the Gal4 activation domain (AD) and binding domain (BD), respectively. Photos were taken after four days of growth on a selective drop-out medium

Importantly, expression of the *PtSmEb* coding sequence under the control of the *p35S* promoter rescued the pleiotropic and cold-sensitive phenotype of independently transformed *Arabidopsis* *sme1-1/pcp-1* lines completely (*n* = 20) or partially (*n* = 29), with only two lines showing no complementation (Fig. 2A-B). Furthermore, yeast-2-hybrid analysis confirmed that PtSmEa and PtSmEb interacted with PtSmGs and PtSmF, as expected for components of the evolutionary conserved Sm ring (Fig. 2C) [18]. Taken together, our data confirmed that *Potra2n6c13821* and *Potra2n18c32411* encode for two identical *P. tremula* SmE proteins.

### Stacked mutations of *SmE* genes affect vegetative growth

**Fig. 3:**
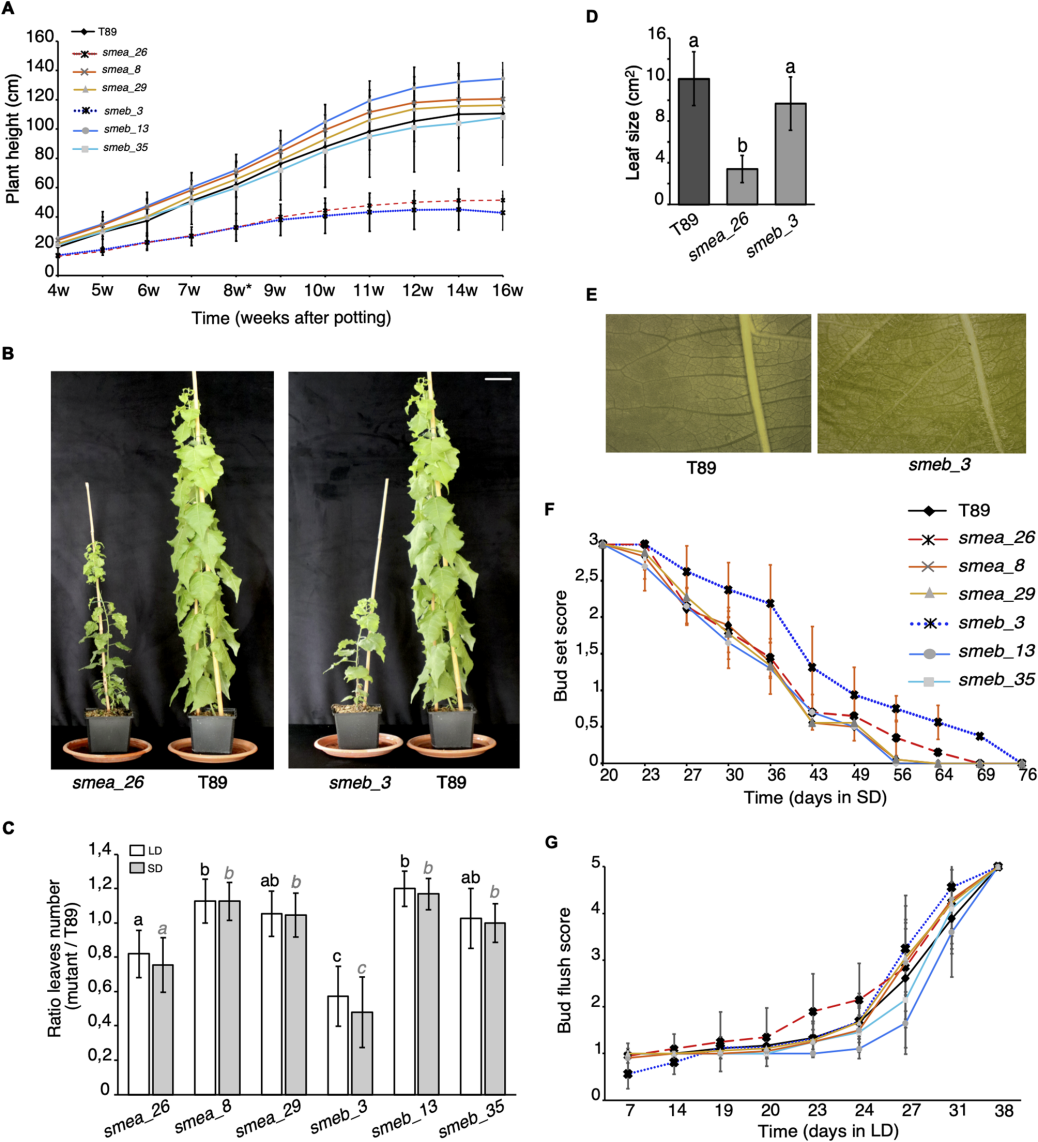
CRISPR/Cas9 lines phenotypes. (**A**) Plant height of the CRISPR/Cas9 lines compared to T89 recorded during the vegetative growth period during LD and SD phases. Error bars indicate s.d. of ten biological replicates. * Indicates end of the LD phase. (**B**) Comparison of T89, *smea_26*, and *smeb_3* mutants at the end of the LD phase. Size bar: 5 cm. (**C**) Ratio of total leaves of mutant plants relative to T89 at the end of the LD and SD seasons. (**D**) Size of the first fully expanded leaf recorded on plants at the end of the LD season (60 DAS) (**E**) Photos show the first fully expanded leaves of WT (T89) and *smeb_3* plants grown under SD for 17 days. (**F**) Bud set score of WT and the six CRISPR/Cas9 trees grown under SD conditions. Scores describe the transition from active growth (3 on the y-axis) to a fully developed bud (0 on the y-axis). Error bars indicate s.d. of 10 biological replicates. (**G**) Bud flush score of WT and the six CRISPR/Cas9 trees after cold treatment and switch to LD season. Scores describe the transition from hard and closed buds (0 on the y-axis) to fully opened buds and growing apices (5 on the y-axis). Error bars indicate s.d. of 10 biological replicates. One-way ANOVA was used for statistical analysis. Data from LD and SD periods were calculated separately in (**C**). Different letters indicate categories that are statistically different (*p* ≤ 0.05)

To investigate the role of the *SmE* genes in tree development, we generated *SmEa* and *SmEb* mutants in hybrid aspen (T89) using CRISPR/Cas9 and pairs of gRNAs specific for each locus (Fig. S2 and Fig. S3). After genotyping several independent lines, three were selected for each gene (Fig. S2). Several mutant alleles, including three knocked-out mutants (*smea_29*, *smeb_3*, and *smeb_13)* were identified and grown in cabinets simulating changes in day length and temperature across the seasons. During the initial growth of eight weeks in long-day conditions (LD^16 h,21 °C^) and the early phase of the following growth in short-day (SD^10 h,21 °C^) from week nine to week 27, *smea_26* and *smeb_3*, grew significantly smaller, set fewer leaves, and responded more rapidly to the photoperiod change by ceasing growth when compared to T89 (Fig. 3A-C). However, despite their overall similar appearance, the size of the first fully expanded leaves was only significantly reduced specifically in *smea_26* when compared to T89 whereas leaves of *smeb_3* were covered with both adaxial and abaxial trichomes (Fig. 3D-E). During the SD^10 h,21 °C^ phase, plants stopped growing, and *smeb_3* plants displayed a slightly delayed bud set (Fig. 3F). None of the other lines showed any significant alterations in vegetative growth. Furthermore, none of the six CRISPR/Cas9 lines showed any differences in bud flush after being exposed to 4 °C for seven weeks (weeks 28–34) and subsequent growth at LD^16 h,21 °C^, suggesting that low temperatures play no or only a very minor effect in the development of *sme* mutants in aspen (Fig. 3G).

**Fig. 4:**
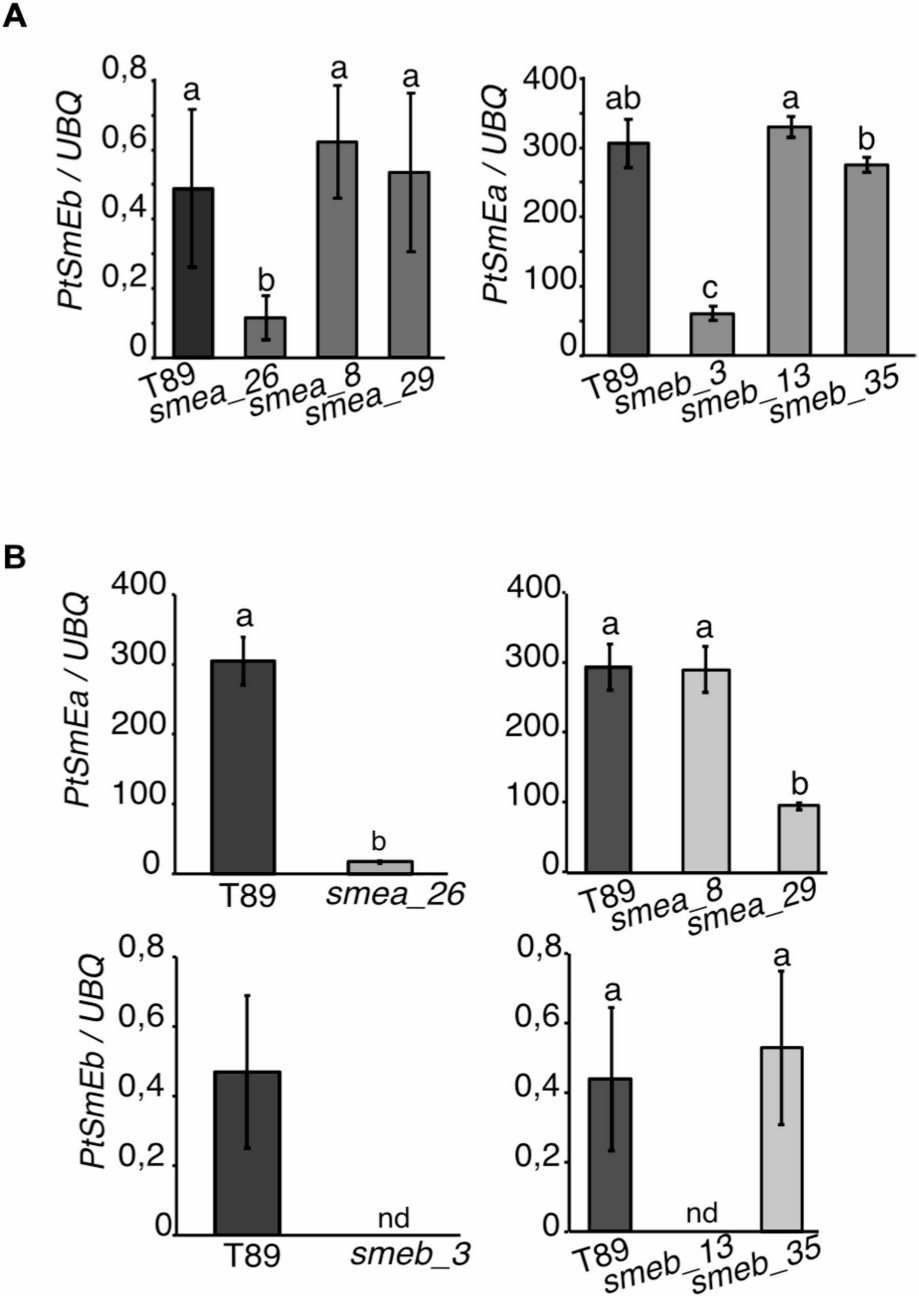
*Sm* expression in CRISPR/Cas9 lines (**A**) Expression of *PtSmEa* and *PtSmEb* in leaves from T89 and CRISPR/Cas9 lines. Error bars indicate s.d. of three biological replicates. (**B**) Expression of *PtSmEa* and *PtSmEb* in leaves from T89 and CRISPR/Cas9 lines. The expression of *SmEa* and *Smeb* was determined by RT-qPCR. Error bars indicate the s.d. of three biological replicates. One-way ANOVA was used for statistical analysis. Different letters indicate categories that are statistically different (*p* ≤ 0.05)

To understand why *smea_26* and *smeb_3* but not *smea_29* and *smeb_13*, which cause large homozygous deletions in the respective genes, displayed developmental phenotypes, we analysed *PtSmE* gene expression in the mutant lines (Fig. 4A-B). Interestingly, we found that among the three CRISPR/Cas9 lines that carried mutations in *PtSmEa*, *smea_26* showed significant downregulation of the paralog *PtSmEb* (Fig. 4A). Vice versa, *smeb_3* was the only *PtSmEb* mutant with reduced *PtSmEa* expression (Fig. 4A). Importantly, whole genome sequencing (WGS) detected no off-target mutations in *smea_26* and *smeb_3* compared to the T89 reference genome. WGS further confirmed the absence of wild-type *PtSmEb* sequences in *smeb_3* but detected three *PtSmEa* alleles, including wild-type *PtSmEa*, in *smea_26*. This result suggested that *smea_26* is a chimeric line or a genetic mosaic, explaining the residual *SmEa* expression detected in the mutant (Fig. 4B). These findings indicate that poplar tolerates the loss of either of the two *SmE* genes, and phenotypes only manifest if the expression of the paralogous gene is reduced by a yet unknown mechanism. Supporting this interpretation, we only recovered lines (*n* = 30) that were either heterozygous or wild-type at the *PtSmEa* locus when we introduced the sgRNAs used to generate *smea_26* into the *smeb_3* background to produce a *SmE* double mutant. These data suggest that, as in metazoan and *Arabidopsis*, complete loss of *SmE* function is lethal in aspen and indirectly suggests that expression of either gene above a minimal threshold is required for plant survival [19–21].

### Transcriptomic confirms *SmEs* role in RNA regulation

**Fig. 5:**
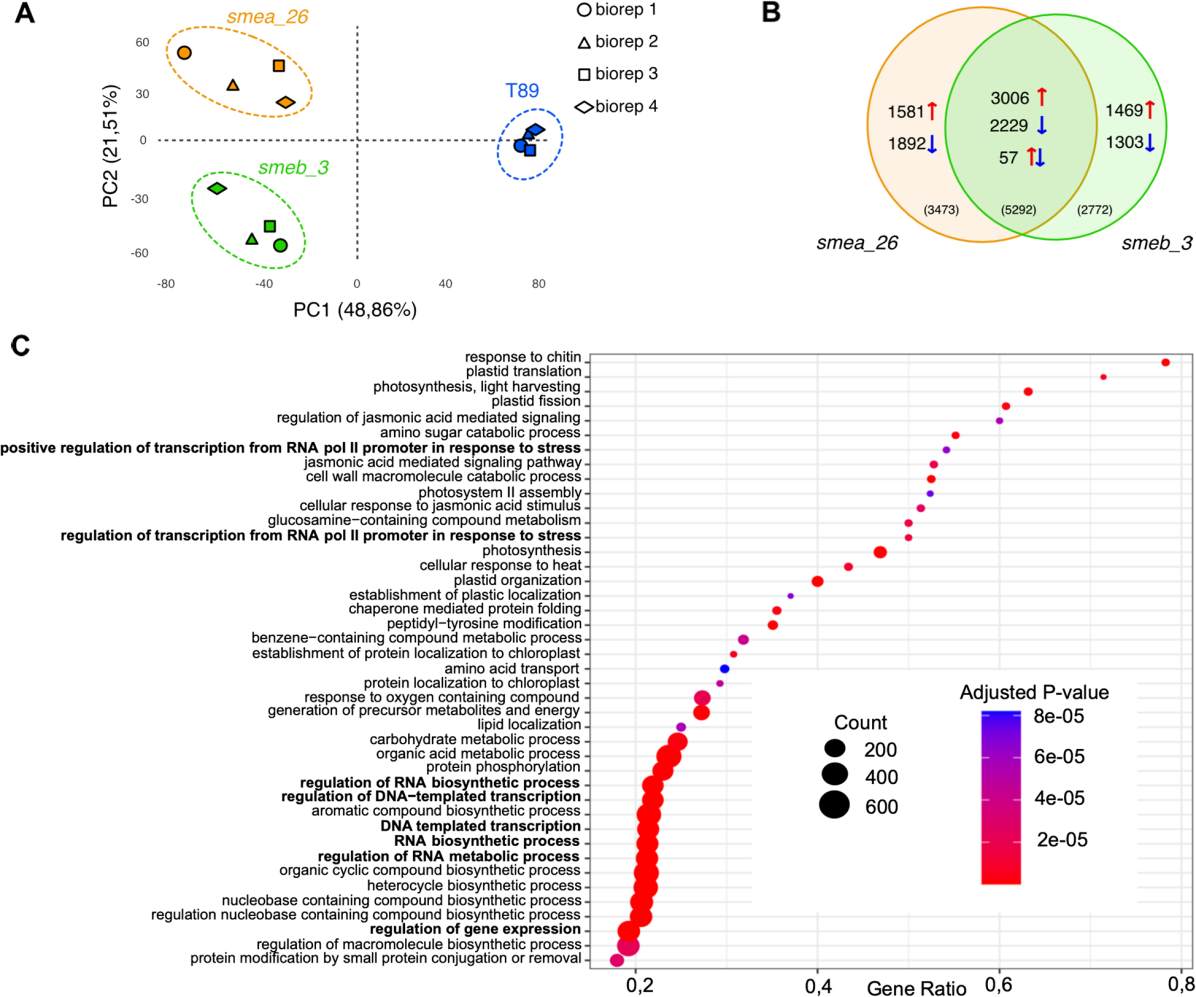
*SmEa* and *SmEb* share the activation of stress-related genes but maintain specific functions (**A**) Principal Component Analysis (PCA) of gene expression in poplar. RNA was extracted from the first true leaf at the end of the LD growth phase, just before the shift to SD photoperiod. Each point represents a biological replicate (*n* = 4). The first two components are plotted, and percentages of variation explained are shown on the axis. (**B**) Venn diagram showing common and exclusive DEGs between *smea_26* and *smeb_3*. Arrows indicate the direction of the modulation, red = up and blue = down. (**C**) GO enrichment analysis of common DEGs between *smea_26* and *smeb_3*. Significantly enriched GO categories were identified by the Benjamini-Hochberg procedure (*P*-value < 0.01)

To investigate the molecular consequences of reduced SmE in poplar, we next performed RNA-sequencing on the first fully expanded leaves collected from trees at the end of the LD^16 h, 21 °C^ growth phase. Principal component analysis showed that the first component separated mutants from wild-type and explained 48.9% of the variance, whereas the second component separated the two mutants (Fig. 5A). Further analysis detected 8765 and 8064 differentially expressed (DE) genes in *smea_26* and in *smeb_3*, respectively, when compared to T89, of which 5292 were shared between the mutants (Fig. 5B; Table S2-S4). Of the latter, 3006 genes were upregulated, and 2229 genes were downregulated in both mutants. The reliability of RNA-seq data was validated by RT-qPCR (Fig. S4). GO analysis on the shared DE genes revealed enrichment in categories related to RNA metabolism and biosynthesis. Genes in these categories are mostly upregulated (Fig. 5C, Table S5, Fig. S5) and include orthologs of pre-mRNA splicing factors and regulators of RNA polymerase II (RNA Pol II) transcription elongation. Furthermore, GO analysis also indicated the activation in both mutants of genes involved in plant defence and responses to jasmonic acid (JA) as well downregulation of photosynthesis (Fig. 5C, Table S5, Table S6, Fig.S5).

Considering the phenotypic differences between the mutants we also inspected genes that were specifically DE in either *smeb_3* or *smea_26*. In *smeb_3*, genes were enriched for GO categories related to protein, carbohydrate metabolism, and RNA biosynthesis (Table S7-12, Fig. S6). In contrast, GO analysis of the 3473 genes that were exclusively DE in *smea_26* revealed significant enrichment in categories that point to a role in the regulation of cell cycle and DNA replication (Table S7-12, Fig. 5C, Fig. S7). Most of the genes in these categories are downregulated in *smea_26*, correlating with the downregulation of genes involved in cell division and suggesting that the reduced growth of the mutant is in part due to impaired cell cycle progression (Fig. 3A-D, Table S9).

Taken together, our phenotypic and transcriptome analyses point to a role for *PtSmEa* and *PtSmEb* in modulating the entire transcriptome, by affecting transcription, splicing and RNA metabolism and ultimately the vegetative growth in trees, with a function of the two genes that is not fully redundant.

### *SmEs* mutation impacts on RNA splicing

**Fig. 6:**
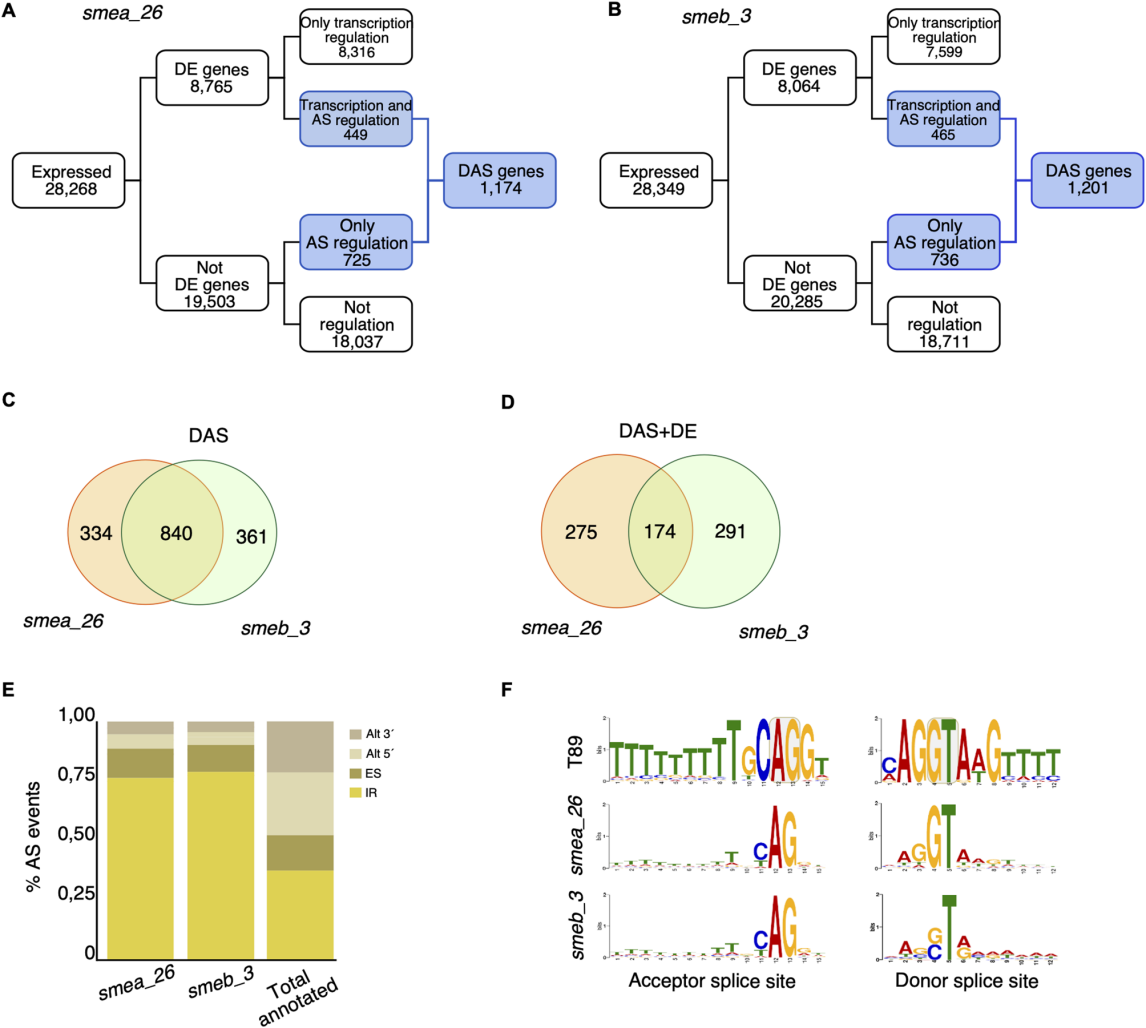
Alternative splicing analysis in *smea_26* and *smeb_3*. (**A**-**B**) Summary figures showing the number of genes and transcripts that are only regulated by transcription (DE), only by alternative splicing (AS), or both transcription and alternative splicing (DAS) in the two mutants. (**C**) Venn diagram showing common and exclusive DAS genes between *smea_26* and *smeb_3*. (**D**) Venn diagram showing common and exclusive DAS + DE genes between *smea_26* and *smeb_3*. (**E**) Frequency of different types of alternative splicing (AS) events. Alt 3´, alternative acceptor site; Alt 5´, alternative donor site; ES, exon skipping; IR, intron retention. (**F**) Pictograms showing the frequency distribution at the acceptor and donor splice sites in wild-type and the two mutants. The relative heights of letters correspond to frequencies of bases at each position and reflect the degree of sequence conservation indicated in bits of information

Given the DE of many genes involved in RNA-related processes and since SmE is a key component of the splicing machinery we analysed our RNA-seq data also for alternative splicing (AS) events compared to the reference transcriptome. In both mutants, we detected slightly more than a thousand differentially alternatively spliced (DAS) genes. Of these, about 70% are shared, indicating partial redundancy in splicing regulation for *SmEa* and *SmEb* (Fig. 6A-C), and 174 genes are both DE and DAS in the two mutants (Fig. 6D, Table S13). In *smeb_3*, DAS genes were enriched for various GO categories, including protein-DNA complex formation and mRNA processing (Fig. S8, Table S14), while s*mea_26* showed enrichment of GO categories related to mRNA metabolism and processing, mismatch repair mechanisms, DNA catabolism, and DNA conformation change. (Fig. S8, Table S15). Furthermore, *smea_26* and *smeb_3* show a similar distribution of different AS events, with intron retention (IR) accounting for about 75% of the overall detected events and a significant underrepresentation of alternative 3´ and 5’ splice sites (Fig. 6E, Table S16, Table S17). Interestingly, the sequences flanking the 5´ and 3´ splice sites of the introns retained in *smea_26* and *smeb_3* were overall rather poorly conserved when compared to the 5’-donor and 3’-acceptor splice sites of 467,737 introns annotated in the T89 genome (Fig. 6F). Since *PtSmE* is a component of the major spliceosomes, it is possible that a mutated U1 snRNP affects 5’ splice site recognition with downstream effects in the splicing cascade, including altered 3´splice site selection.

Overall, these data provide evidence that mutation in *PtSmE* genes can be considered as trans-acting mutations, that directly affect gene expression and splicing across many different pre-mRNA in poplar, thereby causing growth abnormalities.

## Discussion

Sm proteins are core components of snRNP complexes that are usually encoded by two or more paralogous genes in plants [22]. Their role in plant development started to emerge only recently, but analyses have largely been restricted to annual plants [2, 20, 23]. To rectify this situation, here we analysed the role of *SmE* genes in the growth of European aspen. Contrary to reports in *Arabidopsis*, where the single *sme1-1/pcp-1* mutant resulted in strong growth defects, we only detected phenotypes in CRISPR/Cas9 lines, *smea_26* and *smeb_3*, in which expression of both *PtSmE* genes was impaired [2, 3, 5, 24]. As the two poplar *SmE* genes encode the same protein and exhibit similar expression patterns they appear to have redundant functions, as supported by our RNA-seq analyses. This redundancy is further confirmed by the phenotype of CRISPR/Cas9 lines, which only show stunted growth when both genes are misregulated. We speculate that the strong phenotypes observed in the *smea_26* and *smeb_3* lines emerge when both genes are knocked down/out in specific tissues or cell types, or at particular stages of development. Furthermore, since double *PtSmE* knockout mutants could not be recovered and are presumably lethal, our results suggest that like in *Arabidopsis*, a minimal threshold for *PtSmE* mRNA or protein expression exists below which plants are no longer viable.

During vegetative growth, mutation of *PtSmE* induced the aberrant phenotypes described above, as well as changes in expression and splicing of genes involved in various pathways. Our analysis showed that both mutants have an altered distribution of AS events, with an increase in intron retention (IR) events. When unspliced introns remain in mature mRNAs, they are likely to introduce premature stop codons, thereby triggering the nonsense-mediated decay (NMD) pathway, which degrades aberrant mRNAs [25]. NMD plays an important role in regulation of gene expression and is tightly linked to AS. Our data suggest that DE genes in *smea_26* and *smeb_3* are modulated by a direct effect on transcription regulators and by mRNA decay.

Among DE genes, RNA-seq analysis showed changes in expression of genes involved in various RNA processing pathways, such as the orthologs of *Saccharomyces cerevisiae CEF1(CEREVISIAE HOMOLOG OF CDC FIVE)* and *CLF1* (*CROOKED NECK LIKE FACTOR1*), which are core components of the NineTeen Complex (NTC) and participate in the spliceosome catalytic activation [26–29]. NTC has also been implicated in regulating transcription by recruiting accessory factors to RNA Pol II [30–32]. Notably, both poplar CRISPR/Cas9 mutants showed upregulation of genes involved in transcription elongation by RNA Pol II, including *GTA02* (*GLOBAL TRANSCRIPTION FACTOR GROUP A2*), *SPT16* (*GLOBAL TRANSCRIPTION FACTOR C*) and others.

Other GO categories shared between *smea_26* and *smeb_3* notably include the downregulation of genes involved photosynthesis. As a reduced photosynthetic efficiency would provide less energy to the plants, we can speculate that the stunted growth observed in the two mutants may result from a deficit in photosynthetic products such as ATP or NADPH [33–35]. Adjustment in photosynthetic efficiency is of key importance to allow plants to adapt and respond to changing environmental conditions and to abiotic or biotic stressors [36, 37]. Future experiments will need to determine whether the upregulation of stress-related genes observed in *smea_26* and *smeb_3* is a direct consequence of reduced SmE function or indicates genuine stress experienced by the mutants. In either case it is interesting to note that the stunted shoot growth observed in Arabidopsis *sme1-1/pcp-1* mutants has also been attributed to the activation of stress-response mechanisms [5].

Although growth is reduced in both *smea_26* and *smeb_3*, only *smea_26* plants also have significantly smaller first leaves when compared to T89, somewhat reminiscent of the leaf phenotype of *Arabidopsis* *sme1-1/pcp-1*. RNA-seq results suggest that the reduced growth in the first fully expanded leaf might be a consequence of the downregulation of genes involved in cell division, possibly due to reduced expression of cell cycle and DNA replication genes [38, 39]. These findings are similar to the situation in *Arabidopsis*, in which *sme1-1/pcp-1* phenotype has been attributed to downregulation of mitotic markers in roots, suggesting that *SmE* genes might regulate similar processes in the two species [6].

However, contrary to the situation in *Arabidopsis*, where low temperature strongly enhances the phenotype of the *sme1-1/pcp-1*, vegetative growth of *smea_26* and *smeb_3* is strongly affected even under standard growth temperature, and these phenotypes are not exacerbated by lower temperature.

Overall, our results provide a first glimpse into the function of a core splicing protein in the regulation of growth in a perennial species. Although important parallels are identified with *Arabidopsis*, our data indicate specific characteristics of *SmE* in poplar, highlighting the importance of studying those physiological and evolutionary aspects that distinguish this gene family in woody and herbaceous plants.

## Conclusion

Our study provides the first functional characterization of *SmE* genes in a perennial species, offering insights into how core splicing factors contribute to growth regulation in poplar. Unlike *Arabidopsis thaliana*, where mutation of a single *SmE* gene (*sme1-1/pcp-1*) results in severe developmental defects, phenotypic alterations in poplar were only observed when expression of both paralogs was impaired. This indicates a high degree of functional redundancy between *PtSmEa* and *PtSmEb*, supported by their identical protein sequences and overlapping expression profiles. The failure to recover double knockout lines further suggests that a minimal threshold of *SmE* expression is essential for viability, consistent with findings in *Arabidopsis*. Transcriptomic analyses revealed widespread misregulation of genes linked to RNA metabolism, spliceosome activation, transcriptional control, and photosynthesis. These alterations likely underlie the stunted growth observed in the CRISPR/Cas9 mutants. Notably, despite broad similarities with *Arabidopsis*, our data point to distinct features of SmE function in poplar, including growth impairment under standard conditions rather than only at low temperatures. Together, these findings highlight the evolutionary conservation and divergence of *SmE* gene function across herbaceous and woody plants, emphasizing the importance of studying splicing components in perennial species to better understand their roles in growth, development, and stress resilience.

## Supplementary Information

Supplementary Material 1

Supplementary Material 2

## Data Availability

The RNA-seq and WGS data is accessible at the ENA (https://www.ebi.ac.uk/ena/browser/home) under the accession PRJEB86435.
