# Supplementary material for: The splicing genes SmEa and SmEb regulate plant development during vegetative growth in poplar
Source: BMC Plant Biol. 2025 Dec 23;25:1723. doi: 10.1186/s12870-025-07676-3 (PMC12729064; doi:10.1186/s12870-025-07676-3)
Supplement: Supplementary file 1 — Supplementary Material 1 [file 12870_2025_7676_MOESM1_ESM.pdf]

The splicing genes *SmEa and SmEb* regulate plant development during vegetative growth in poplar

Goretti D., Collani S., Marcon A., Nilsson O., Schmid M.

The following Supplementary Figures and Tables are available for this article:

Fig. S1 SmE proteins from Arabidopsis and poplar are highly conserved.

Fig. S2 Description of the CRISPR/Cas9 lines.

Fig. S3 Nucleotide diversity at *SmEa* and *SmEb* from *P. tremula.*

Fig. S4 Validation of RNA-seq by RT-qPCR.

Fig. S5 GO enrichment analysis of common up/down DEGs between *smea_26* and *smeb_3*.

Fig. S6 GO enrichment analysis of DEGs specific to *smeb_3*.

Fig. S7 GO enrichment analysis of DEGs specific to *smea_26*.

Fig. S8 GO enrichment analysis of DAS in *smeb_3* and *smea_26*.

Table S1 *Sm* and *LSm* genes in different species.

Table S2 List of DEGs in *smea_26*.

Table S3 List of DEGs in *smeb_3*.

Table S4 List of DEGs common to *smea_26* and *smeb_3.*

Table S5 List of upregulated DEGs common to *smea_26* and *smeb_3*.

Table S6 List of downregulated DEGs common to *smea_26* and *smeb_3*.

Table S7 List of DEGs specific for *smea_26*.

Table S8 List of upregulated DEGs specific for *smea_26*.

Table S9 List of downregulated DEGs specific for *smea_26.*

Table S10 List of DEGs specific for *smeb_3*.

Table S11 List of upregulated DEGs specific for *smeb_3*.

Table S12 List of downregulated DEGs specific for *smeb_3*.

Table S13 List of common and specific DE and DAS genes.

Table S14 GO analysis in *smeb_3*.

Table S15 GO analysis in *smea_26*.

Table S16 AS events in *smeb_3.*

Table S17 AS events in *smea_26*.

Table S18 List of oligos used in this study.

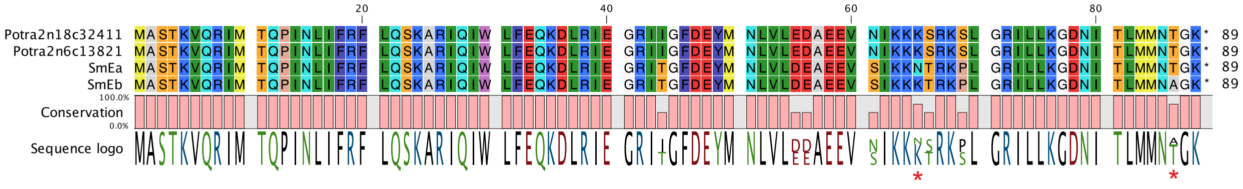

Fig. S1 SmE proteins from Arabidopsis and poplar are highly conserved. Alignment of SmE proteins from Arabidopsis (SmEa and SmEb) and *Popolus tremula.* Red stars indicate non-synonymous amino acid substitutions between the two Arabidopsis SmE proteins.

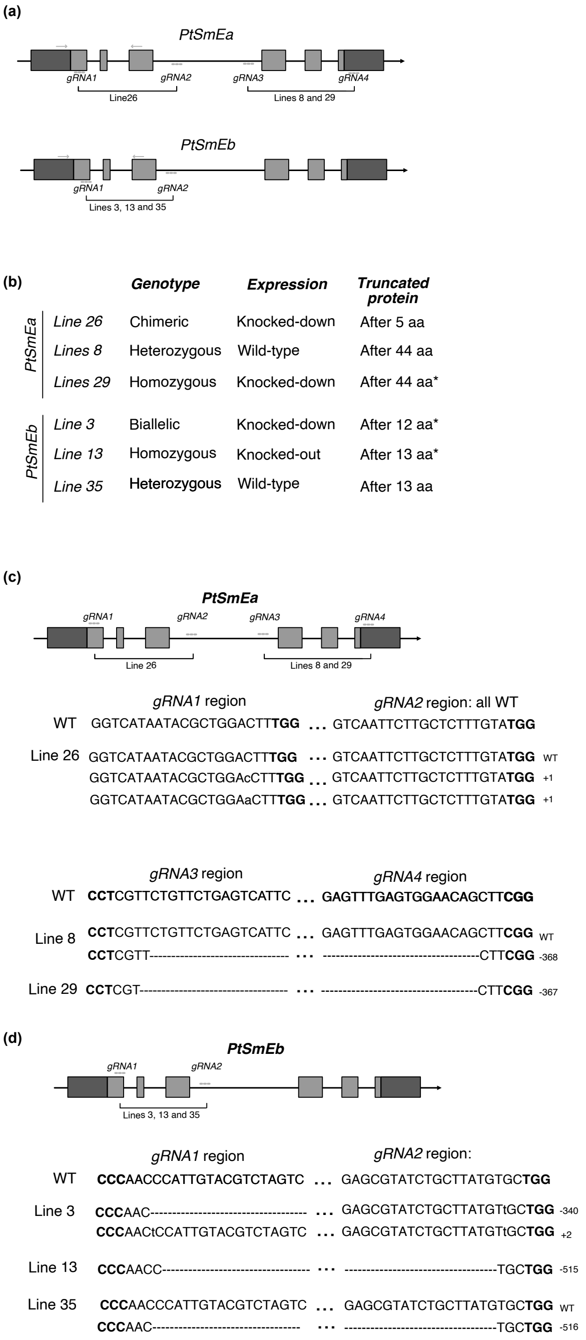

Fig. S2 Description of the CRISPR/Cas9 lines. (a) Schematic representation of *PtSmEa* and *PtSmEb* loci in *P. tremula*. The position of gRNAs used to generate each CRISPR/Cas9 line is indicated. (b) Effect of the CRISPR/Cas9 mutagenesis on the phenotype, indicated as gene expression level and predicted effect on the SmE protein sequence. (c-d) Sequence of CRISPR/Cas9 alleles compared to wild-type. Deletion or insertion length is indicated on the right side. Bold characters indicate the PAM sequence.

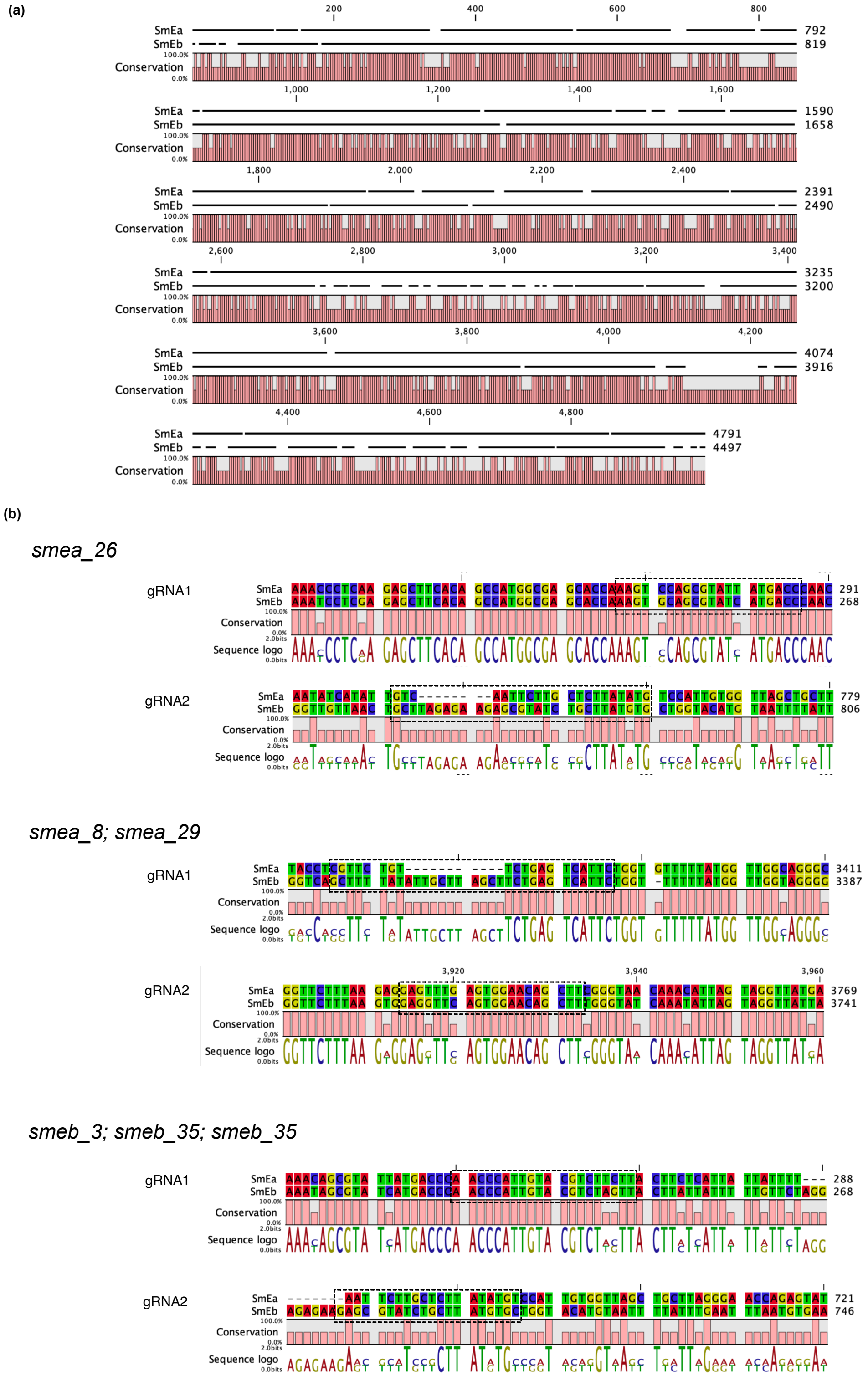

Fig. S3 Nucleotide diversity at *SmEa* and *SmEb* from *P. tremula*. (a) Alignment of genomic *SmEa* and *SmEb* loci. Bars indicate the conservation of each nucleotide position. (b) Alignment of genomic *SmEa* and *SmEb* region covering the gRNA sites.

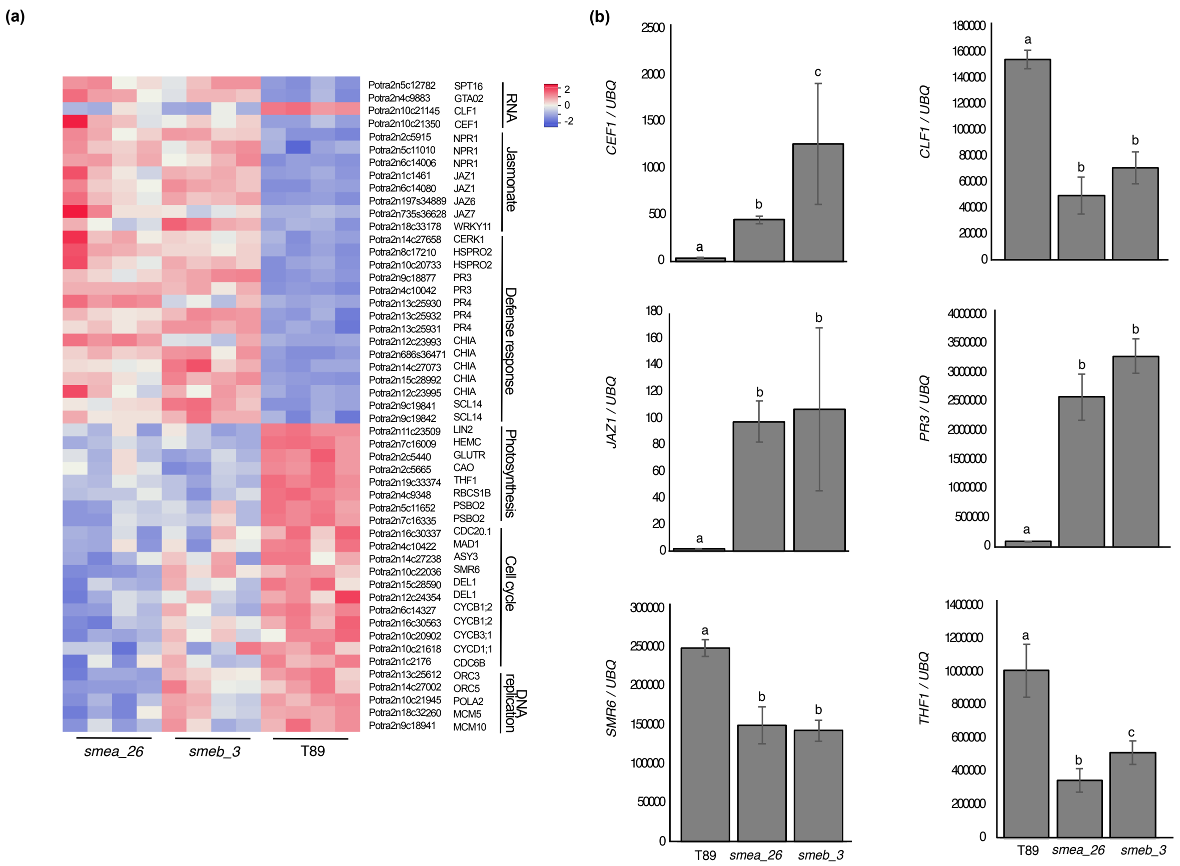

Fig. S4 Validation of RNA-seq by RT-qPCR. (a) Heatmaps of DEGs in first fully expanded leaves from *smea_26*, *smeb_3*, and wild-type plants organised according to their function. Shades of red and blue indicate up- and down-regulated genes, respectively. The short gene name of the Arabidopsis homolog is shown next to each *P. tremula* locus. (b) Expression in *smea_26*, *smeb_3* and T89 leaves of some genes reported in (a). Expression was determined by RT-qPCR. Error bars indicate the s.d. of three biological replicates.

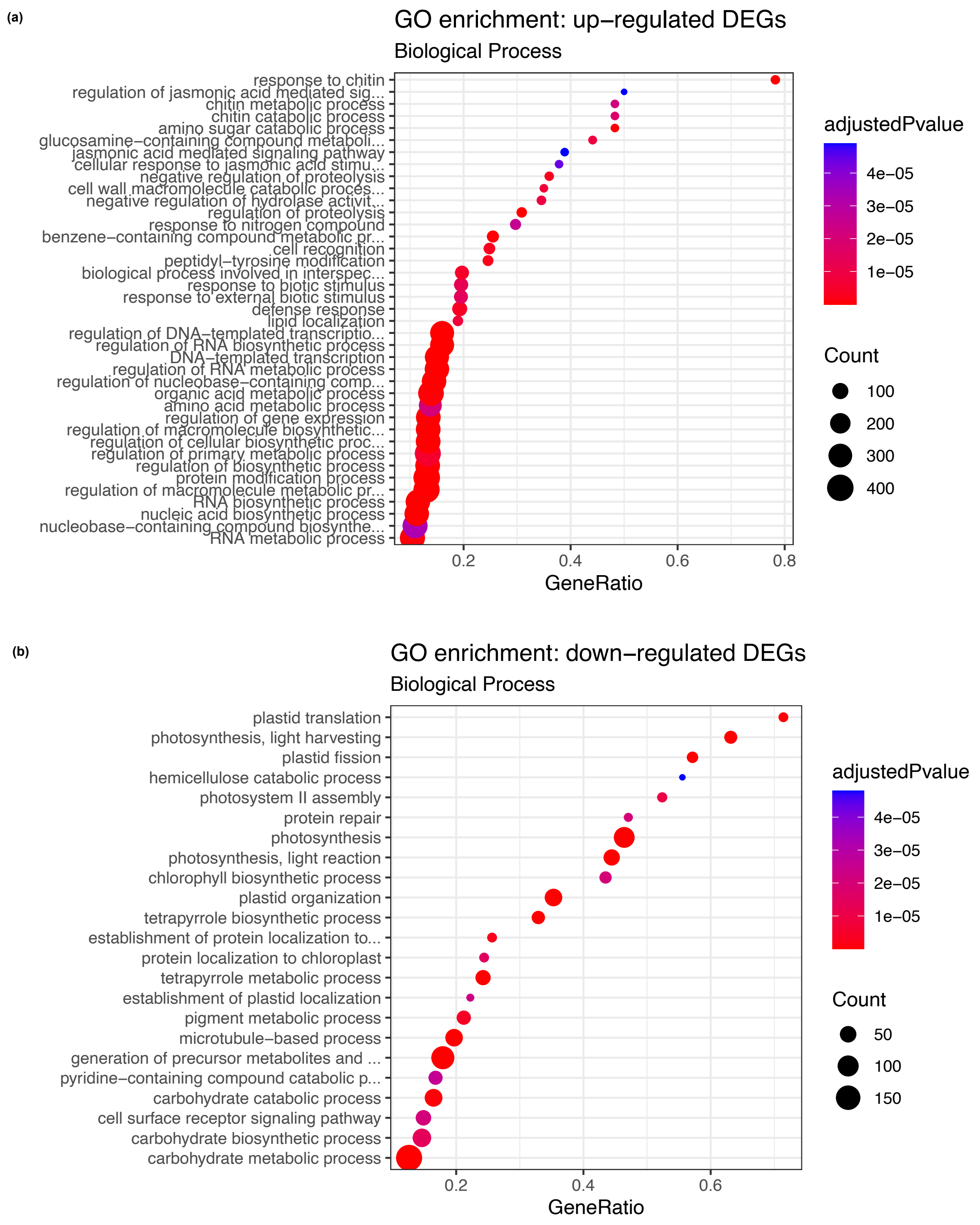

Fig. S5 GO enrichment analysis of common up/down DEGs between *smea_26* and *smeb_3*. Significantly enriched GO categories were identified by the Benjamini-Hochberg procedure (P-value < 0.01). (a) GO enrichment for genes up-regulated in *smea_26* and *smeb_3*. (b) GO enrichment for genes down-regulated genes in *smea_26* and *smeb_3*.

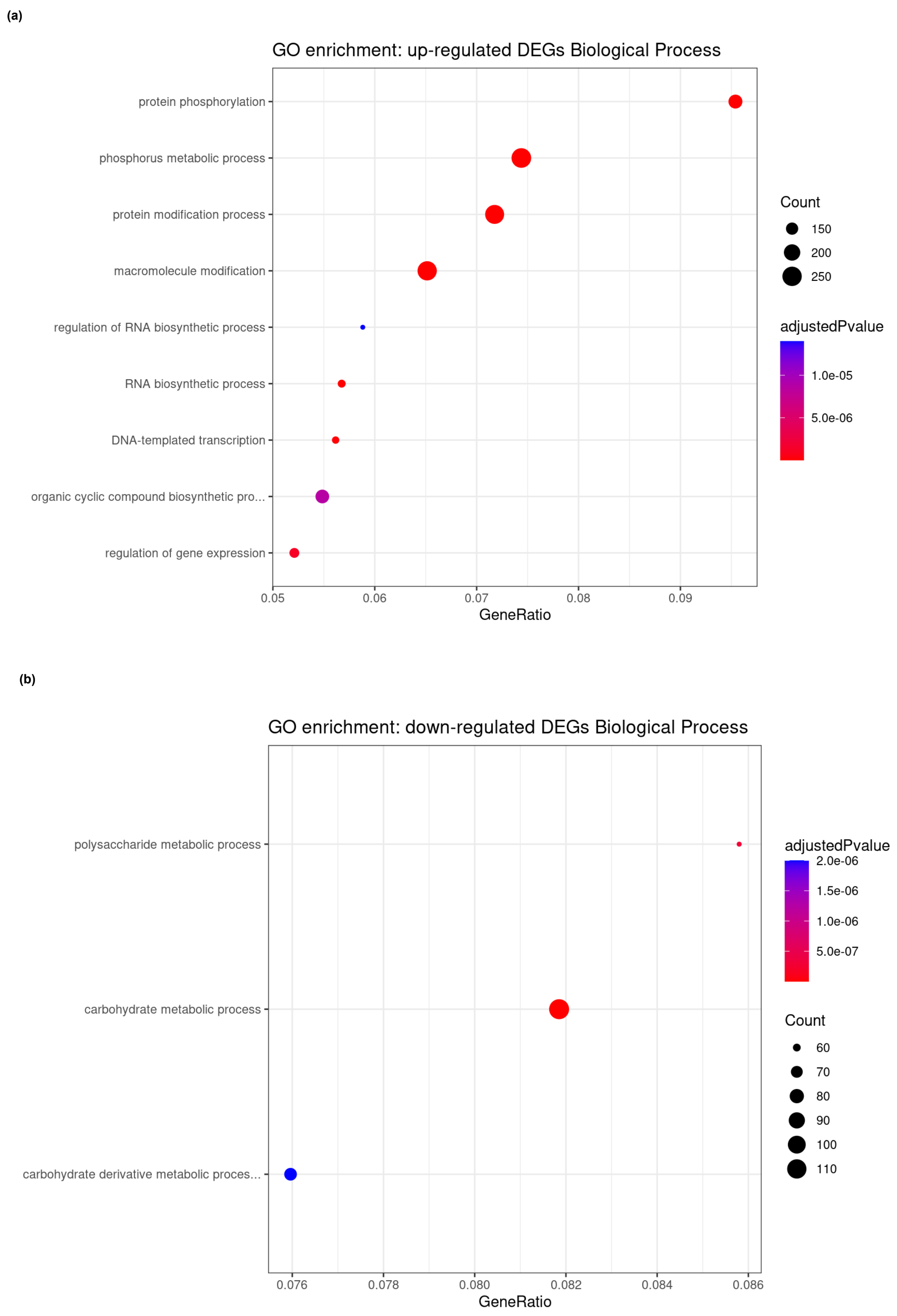

Fig. S6 GO enrichment analysis of DEGs specific to *smeb_3*. Significantly enriched GO categories were identified by the Benjamini-Hochberg procedure (P-value < 0.01). (a) GO enrichment for genes up-regulated in *smeb_3*. (b) GO enrichment for genes down-regulated in *smeb_3*.

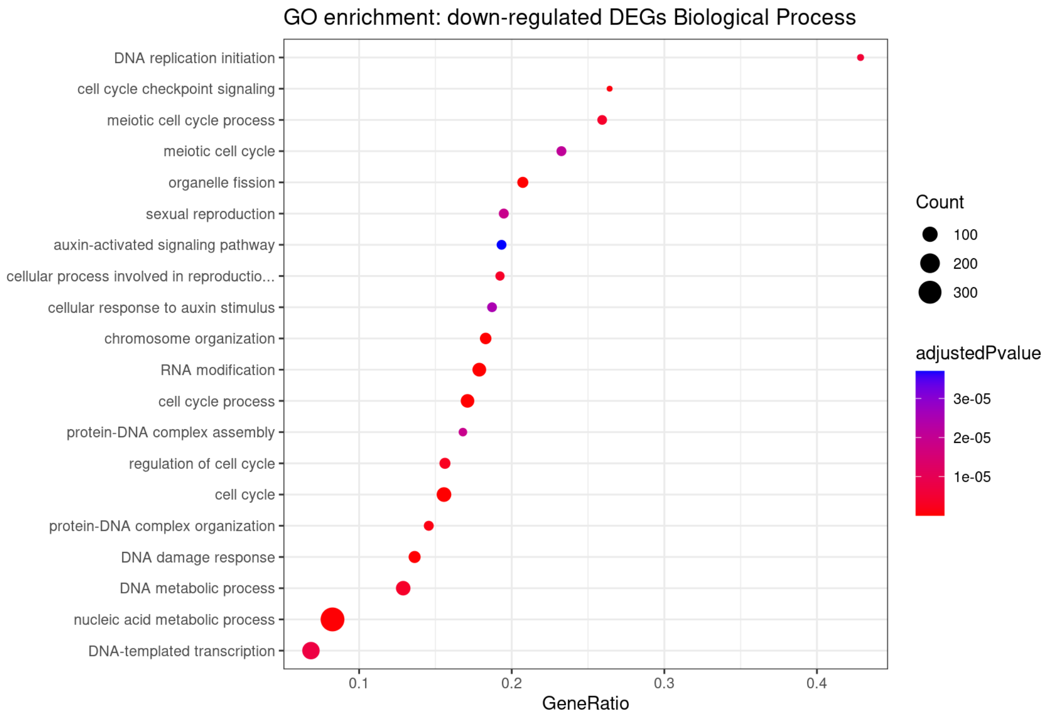

Fig. S7 GO enrichment analysis of DEGs specific to *smea_26*. Significantly enriched GO categories were identified by the Benjamini-Hochberg procedure (P-value < 0.01). GO enrichment was calculated only for genes down-regulated in *smeb_26*.

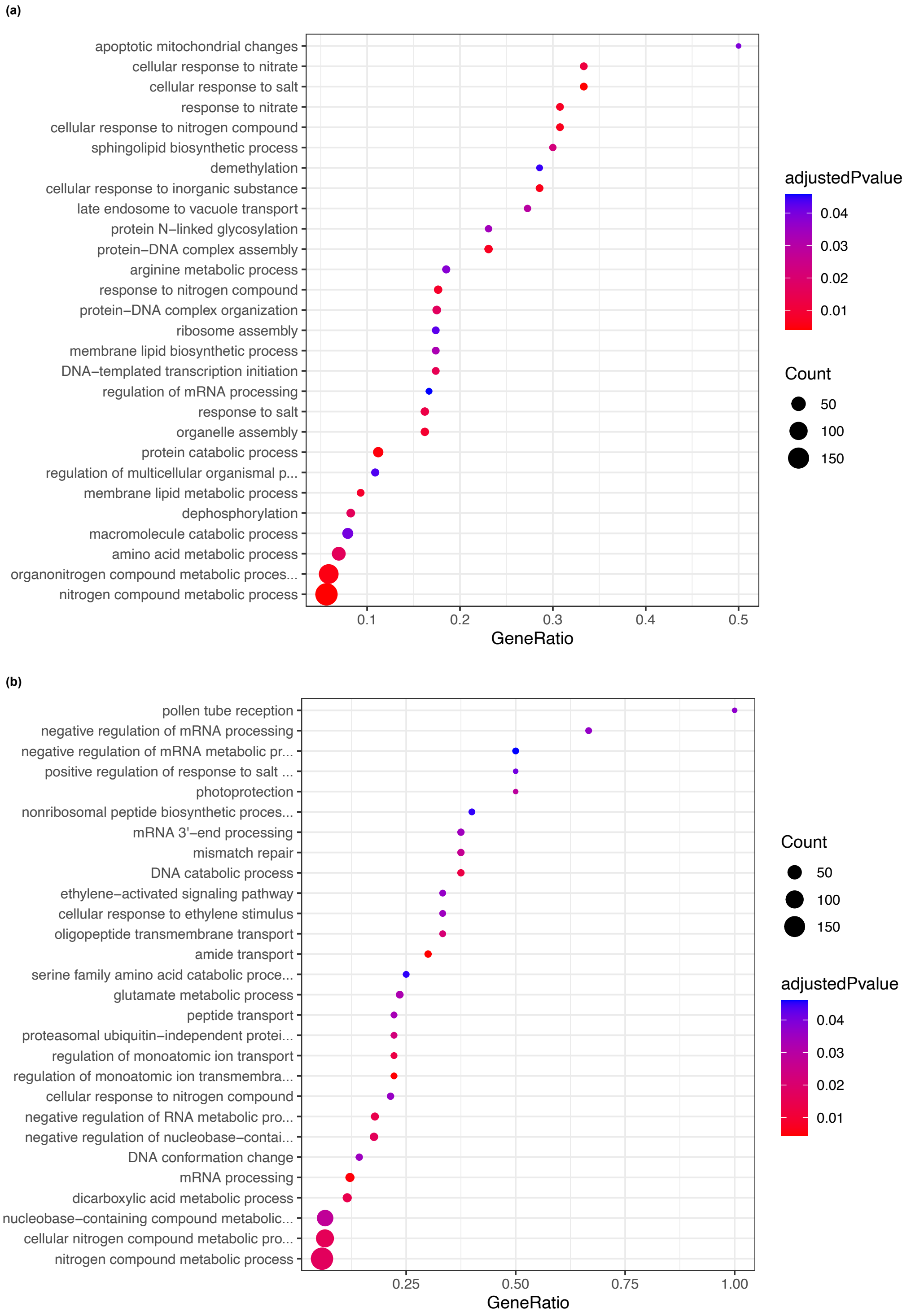

Fig. S8 GO enrichment analysis of DAS in *smeb_3* and *smea_26*. (a) GO categories overrepresented in *smeb_3*. (b) GO categories overrepresented in *smea_26*. Significantly enriched GO categories were identified by the Benjamini-Hochberg procedure (P-value < 0.01).
